# Pre-fractionation strategies to resolve pea (*Pisum sativum*) sub-proteomes

**DOI:** 10.3389/fpls.2015.00849

**Published:** 2015-10-19

**Authors:** Claudia-Nicole Meisrimler, Ljiljana Menckhoff, Biljana M. Kukavica, Sabine Lüthje

**Affiliations:** ^1^Oxidative Stress and Plant Proteomics Group, Biocenter Klein Flottbek and Botanical Garden, University of HamburgHamburg, Germany; ^2^Laboratoire de Biologie du Développement des Plantes, CEA, IBEBSaint-Paul-lez-Durance, France; ^3^Centre National de la Recherche Scientifique, UMR 7265 Biologie Vegetale et Microbiologie EnvironnementalesSaint-Paul-lez-Durance, France; ^4^Aix Marseille Université, BVME UMR7265Marseille, France; ^5^Faculty of Science and Mathematics, University of Banja LukaBanja Luka, Bosnia and Herzegovina

**Keywords:** *Pisum sativum*, cell wall, apoplast, plasma membrane, chloroplast, mitochondria

## Abstract

Legumes are important crop plants and pea (*Pisum sativum* L.) has been investigated as a model with respect to several physiological aspects. The sequencing of the pea genome has not been completed. Therefore, proteomic approaches are currently limited. Nevertheless, the increasing numbers of available EST-databases as well as the high homology of the pea and medicago genome (*Medicago truncatula* Gaertner) allow the successful identification of proteins. Due to the un-sequenced pea genome, pre-fractionation approaches have been used in pea proteomic surveys in the past. Aside from a number of selective proteome studies on crude extracts and the chloroplast, few studies have targeted other components such as the pea secretome, an important sub-proteome of interest due to its role in abiotic and biotic stress processes. The secretome itself can be further divided into different sub-proteomes (plasma membrane, apoplast, cell wall proteins). Cell fractionation in combination with different gel-electrophoresis, chromatography methods and protein identification by mass spectrometry are important partners to gain insight into pea sub-proteomes, post-translational modifications and protein functions. Overall, pea proteomics needs to link numerous existing physiological and biochemical data to gain further insight into adaptation processes, which play important roles in field applications. Future developments and directions in pea proteomics are discussed.

## Introduction

Pea (*Pisum sativum* L.) belongs to the legume family (Fabaceae). Two types of pea can be distinguished: garden pea (green pea) and field pea (dry pea), both of which are important crop plants due to their high iron, starch and protein content (Dahl et al., 2012). Health benefits of peas result from their low-fat content, high levels of antioxidants, anti-inflammatory agents, carotenoids, vitamins B and E. Additionally, pea are a reliable source of omega-3 fats (alpha-linolenic acid, ALA) and omega-6 fatty acid (linoleic acid).

Pea appears to have an unusual combination of antioxidant and anti-inflammatory phytonutrients. A recent study showed that daily consumption of green peas lowers the risk of stomach cancer, due to the presence of coumestrol and pea protease inhibitors (Clemente et al., 2012). They also contain saponins that in combination with other pea components may lower the risk of type-2 diabetes.

Symbiosis of pea with nitrogen-fixing bacteria reduces the use of nitrogen fertilizers. In cultivation, rotation of peas with other crops lowers the risk of pest problems. Additionally, the pea root system prevents erosion of the soil.

According to FAOSTAT data (September 2015), world production of the garden pea increased from 4,716,649 t in 1970 to 18,490,920 t in 2012. The top five countries for garden pea production are: China (11,500,000 t), India (3,650,000 t), France (591,100 t), United States (358,560 t), and Egypt (180,631 t).

Since the initial studies by Gregor Mendel, the garden pea became the most-characterized legume. It has been used in numerous investigations in plant biochemistry and physiology. Methods for pea transformation and production of mutants have been established (Grant and Cooper, 2006). Adaptation of pea cultivars and breeding lines (http://www.seedsanctuary.com/peas/index.cfm or http://bioinf.scri.ac.uk/germinate_pea/app/) to environmental conditions and biotic or abiotic stress factors is reflected by their molecular configuration. Thus, knowledge of gene expression, regulation of enzyme activities and alterations in protein profiles will be of importance for production of stress tolerant and resistant legumes in the future.

Due to the importance in field applications and to the human diet, more and more proteome studies on different aspects of pea were published in the last few years. Many proteome studies on pea, but also on model plants, have been undertaken with crude extracts. Although crude extracts provide information on alterations of a proteome under various conditions, low abundant proteins or membrane bound proteins may not be resolved. To overcome these problems especially in non-model plants, cell fractionation and investigation of sub-proteomes, are powerful alternatives. Approaches readily exist to fractionate a variety of sub-proteomes and can be adapted depending on the scientific question.

## Pea as a model for proteomic studies

Several investigations have been presented on alterations of protein profiles of pea under different physiological conditions (Table 1). Proteomic approaches for non-model species, like pea, are currently limited because the identification of peptides critically depends on an available sequence database. In contrast to the model legume *Medicago truncatula* Gaertner, the pea genome is five to ten times larger and not yet sequenced (Kaló et al., 2004). It consists of 4300 megabases with a high number of repetitive elements (Macas et al., 2007). An increasing number of ESTs are available for pea (http://www.comparative-legumes.org/). Next generation sequencing has produced libraries from flowers, leaves, cotyledons, epi- and hypocotyls, and etiolated and light treated etiolated seedlings (Franssen et al., 2011). The high conservation of the pea and medicago genomes (http://www.medicago.org/index.php) allows identification of pea proteins by mass spectrometry (MS).

**Table 1:** **Studies on pea (*Pisum sativum*) sub-proteomes. DIM, detergent insoluble membrane**.

**Organ**	**Experimental background**	**Sub-proteome**	**Method**	**Identification**	**References**
Seed		Crude extract	2D- IEF/SDS	MALDI-Tof MS	1
Seed	Inbred lines	Crude extract	2D- IEF/SDS	Reference map, MALDI-Tof MS	2
Seed	Genetic modification	Crude extract	2D- IEF/SDS	MALDI Tof/Tof MS	3
Seed	Osmotic stress	Crude extract	2D- IEF/SDS	MALDI Tof/Tof MS	29
Seed	Desiccation	Soluble fraction	2D- IEF/SDS	MALDI-Tof-Tof-MS	4
Seed	H_2_O_2_	Soluble fraction	1D, 2D- IEF/SDS	MALDI-Tof-Tof-MS	5
Leaf	*Uromyces pisi*, BTH, BABA	Crude extract	2D- IEF/SDS	MALDI-Tof/Tof-MS	6
Leaf	*Orobanche crenata*	Crude extract	2D-DIGE	MALDI MS/MS	7
Leaf	*Mycosphaerella pinodes*	Crude extract	2D- IEF/SDS	MALDI Tof/Tof MS	8
Leaf	*Erysiphe pisi*	Crude extract	2D- IEF/SDS	MALDI Tof/Tof MS	9
Leaf	Plum pox virus	Soluble fraction	2D- IEF/SDS	MALDI-Tof, ion trap analysis	10
Leaf	Salicylate	Soluble fraction	2D- IEF/SDS	MALDI Tof MS	11
Leaf	Cold acclimation	Soluble fractions	2D- IEF/SDS	ESI MS/MS	12
Leaf	*Peronospora viciae*	Cytoplasmic, membrane, nucleic acid-associated proteins	2D-DIGE	ESI-Q-Tof-MS/MS MALDI-Tof-MS	13
Leaf	Development	Soluble mitochondrial proteins	2D- IEF/SDS, SEC	Edman, MALDI, ESI	14
Leaf		Thylakoid membranes	BN-PAGE, 1D-SDS, 2D-BN/SDS	LC-ESI-QTOF-MS, MALDI Tof/Tof MS	28
Leaf		Chloroplast envelope	1D-SDS	LC-MS	15
Leaf		Soluble chloroplast proteins	SEC, Affinity LC	ESI-MS/MS	16
Leaf		Chloroplast DIM	1D-SDS	LC-MS	17
Leaf		Chloroplast	BN-PAGE	MALDI-Tof-MS	18
Leaf		Chloroplast Grana	LC-MS	ESI-LC-MS	19
Leaf	Cold, drought herbicides	Soluble mitochondrial membranes	2D-IEF/SDS, BN-PAGE	Q-TOF MS	20
Leaf	Development	Etioplast, chloroplast	BN-PAGE	ESI-MS/MS	21
Leaf		Inner, outer chloroplast envelope	2D- IEF/SDS-PAGE LC-MS/MS	LC-MS/MS	30
Leaf	N mobilization	Crude extract	2D- IEF/SDS	ESI-LC MS	22
Root	Salt	Crude extract	2D- IEF/SDS	ESI-Q-Tof-MS/MS	23
Root	*Orobanche crenata*	Crude extract	2DE-IEF/SDS	MALDI-Tof-MS	24
Root	Cold acclimation	Soluble fraction	2D- IEF/SDS	ESI MS/MS	12
Root		Microsomes	Off-gel	MALDI-Tof/Tof-MS	25
Root	-Fe, chitosan	Plasma membrane	2D-DIGE	MALDI-Tof/Tof-MS	26
Root	Symbiosis	Peribacteroid membrane Peribacteroid space fraction	2D- IEF/SDS	ESI-Q-Tof-MS/MS	27
Stem	N mobilization	Crude extract	2D- IEF/SDS	ESI-LC MS	22
Stem	Cold acclimation	Soluble fractions	2D- IEF/SDS	ESI MS/MS	12

*(1) Bourgeois et al. (2009), (2) Bourgeois et al. (2011), (3) Chen et al. (2009), (4) Wang et al. (2012), (5) Barba-Espín et al. (2011), (6) Barilli et al. (2012), (7) Castillejo et al. (2011), (8) Castillejo et al. (2010), (9) Curto et al. (2006), (10) Díaz-Vivancos et al. (2008), (11) Tarchevsky et al. (2010), (12) Dumont et al. (2011), (13) Amey et al. (2008), (14) Bardel et al. (2002), (15) Bräutigam et al. (2008), (16) Bayer et al. (2011), (17) Phinney and Thelen (2005), (18) Ladig et al. (2011), (19) Gómez et al. (2002), (20) Taylor et al. (2005), (21) Kanervo et al., 2008, (22) Schiltz et al. (2004), (23) Kav et al. (2004), (24) Castillejo et al. (2004), (25) Meisrimler and Lüthje (2012), (26) Meisrimler et al. (2011), (27) Saalbach et al. (2002), (28) Pagliano et al. (2014), (29) Brosowska-Arendt et al. (2014), (30) Gutierrez-Carbonell et al. (2014)*.

As shown in Table 1, proteomic approaches have been published for crude extracts of seeds, leaves and roots of pea. In seeds, the identification of high abundance of storage proteins was demonstrated (Bourgeois et al., 2009, 2011; Chen et al., 2009; Dziuba et al., 2014). Proteomic tools have been used in allergen mapping, e.g., some of the pea storage proteins have a high allergenic potential. In leaves, mainly soluble proteins and components of photosynthesis were detected (Barilli et al., 2012; Pagliano et al., 2014). In roots, proteins of the carbohydrate and nitrogen assimilation pathways, proton transporters and components of the respiratory chain were identified (Castillejo et al., 2004; Kav et al., 2004).

A recent study focused on the post-translational modification of lysine by acetylation in mitochondrial pea proteins of 14–17 day old seedlings (Smith-Hammond et al., 2014). In the study, 664 sites of lysine acetylation on 358 proteins were identified. A statistically significant compartment-specific plant acetylation site motif resembling one from mammalian mitochondria was identified and the biological relevance of this post-translational modification in plants has been confirmed.

Protein alterations during nitrogen mobilization from leaves to filling seeds were published for pea. The study by Schiltz et al. (2004) showed a clear correlation between ribulose-1,5-bisphosphate carboxylase/oxygenase (RuBisCO) degradation and the increase in protease abundance supporting the importance of RuBisCO for nitrogen mobilization. RuBisCO, however, is extremely abundant in leaves and hinders the detection of low abundant proteins. Therefore, strategies to deplete RuBisCO in leaf samples have been developed (Krishnan and Natarajan, 2009; Bayer et al., 2011; Aryal et al., 2012).

Several biotic stress factors from altered protein profiles of pea leaves have been characterized (Curto et al., 2006; Amey et al., 2008; Castillejo et al., 2010, 2011; Brosowska-Arendt et al., 2014). The majority of proteins identified after elicitation were metabolic and stress-related proteins. In roots, proteins of carbohydrate and nitrogen metabolism and mitochondrial electron transport chain decreased after treatment with the parasite crenate broomrape (*Orobanche crenata* Forssk., 1775), while proteins that correspond to enzymes of the nitrogen assimilation pathway or typical pathogen defense pathways increased (Castillejo et al., 2004). Abiotic stress factors resulted in alterations to protein profiles of pea. Crude extracts of salt treated roots revealed pathogenesis-related proteins, antioxidant enzymes, including superoxide dismutase and nucleoside diphosphate kinase (Kav et al., 2004).

Another study was focused on seeds of genetically modified peas expressing the gene for alpha-amylase inhibitor-1 (alphaAI1) from the common bean, which exhibits resistance to the pea weevil (*Bruchus pisorum* Linnaeus, 1758). This proteomic analysis compared seeds from the transgenic pea lines expressing the bean alphaAI1 protein and the corresponding alphaAI1-free segregating lines (Chen et al., 2009). The analysis showed that in addition to the presence of alphaAI1, 33 other proteins were differentially accumulated in the alphaAI1-expressing lines. Only three were found to be associated with the expression of alphaAI1, the other 30 remaining proteins appeared to be associated with *Agrobacterium*-mediated transformation events. The authors found out that the identified proteins with altered accumulation in the pea were mostly storage proteins. This observation led to the suggestion that both transgenesis and transformation events lead to demonstrable changes in the proteomes of peas (Chen et al., 2009).

The high abundance of RuBisCO in leaf samples and the limits of detection for low abundant proteins demonstrated the need for sub-proteome analysis. Several studies have been conducted on fractions of soluble proteins, chloroplasts or mitochondria, and a few on microsomes or plasma membranes (Table 1). Proteomes of cell wall fractions or the apoplastic fluid have not yet been investigated for pea thus far.

Most proteome analyses of the chloroplast and mitochondrion have been undertaken in context to plant development. Different native PAGE approaches like histidine- and deoxycholate-based native PAGE, blue native PAGE (BNE) and high resolution clear native PAGE (hrCNE) were compared for chloroplast complexes of pea and other plants (Ladig et al., 2011). The study of Kanervo et al. (2008) investigated alterations of photosystems I and II complexes during the transition of etioplasts to chloroplasts by BN-PAGE. Chloroplast envelope proteomes of C4 and C3 plants were compared by SDS-PAGE in combination with LC-MS/MS (Bräutigam et al., 2008). The data demonstrated specific adaptations in the chloroplast envelope for C4 metabolism. Additionally, the grana proteome of thylakoid membranes was analyzed by LC-MS/MS to verify subunits of photosystem II and to identify their post-translational modifications (PTMs) (Gómez et al., 2002). A membrane-depleted, high-density fraction was isolated from plastids (Triton X-100 insoluble membranes) for the investigation of intact macromolecular structures and for the analysis of protein-protein and protein-nucleic acid interactions (Phinney and Thelen, 2005). Alterations in abundance of uncoupling proteins and non-phosphorylating respiratory pathways have been demonstrated in pea mitochondria by herbicides (Taylor et al., 2005). These changes revealed lipid peroxidation and oxidative modification of lipoic acid moieties. A comparison of soluble mitochondrial proteomes led to the identification of a number of proteins, which were specifically present in the root or in the mitochondria of seeds (Bardel et al., 2002). The data revealed the impact of tissue differentiation at the mitochondrial level.

All these data demonstrate that proteomic approaches are powerful tools to understand the molecular mechanisms of pea development and biotic or abiotic stress responses. Only the most abundant proteins were identified in crude extracts of seeds, leaves or roots. These results show the need for further investigations of the sub-proteomes of the pea secretome (e.g., the plasma membrane, cell wall, apoplastic fluid) and other cellular compartments aside from the chloroplast in the future.

## An example for a pre-fractionation strategy in pea—the secretome

The plant secretome refers to the set of proteins secreted out of the plant cell into the surrounding extracellular space commonly referred to as the apoplast. The plasma membrane separates the apoplast from the symplast (Alexandersson et al., 2013). Similar to apoplastic proteins, plasma membrane proteins are directed through the endoplasmic reticulum/Golgi pathway and can be defined as part of the secretome.

**Figure 1:**
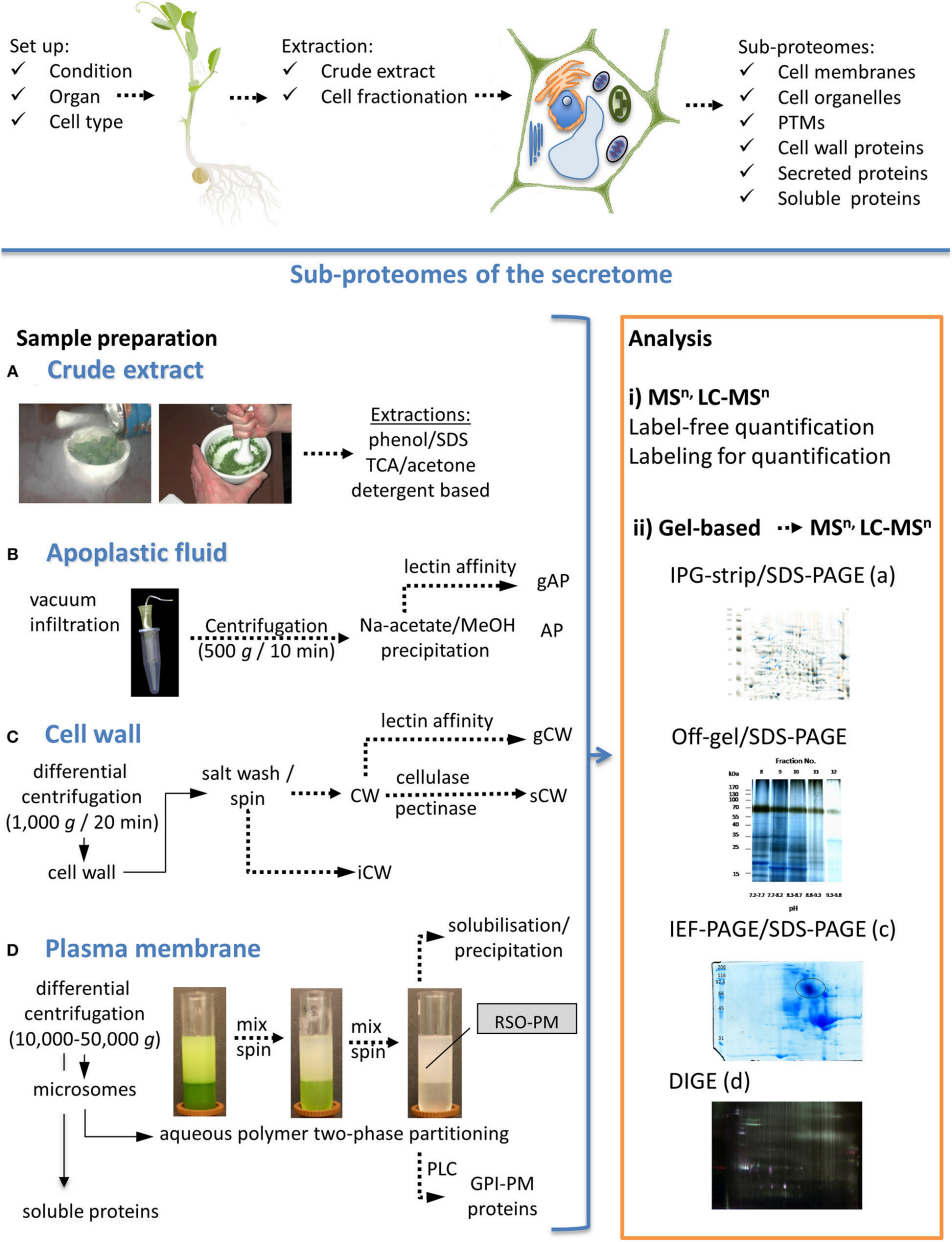
**Pea sub-proteomes with a focus on the secretome**. Scheme for the planning of the experimental set-up and sample preparation is shown on the top. Sample preparation to study proteomes of crude extracts and different sub-proteomes are shown on the left hand, gel-free and gel-based proteome analyses are shown on the right hand. **(A)** Most crude extract preparation protocols are based on solvents, acids and detergents (Giavalisco et al., 2003; Wang et al., 2010; Wu et al., 2014). **(B)** Protocols for preparation of apoplastic fluids (AP) have been presented by Zhou et al. (2011) and Witzel et al. (2011). **(C)** Cell walls (CW) can be isolated according to Kukavica et al. (2012). **(D)** State of the art for plasma membrane (PM) preparation is aqueous polymer two-phase partitioning. Proteomes can be analyzed either by (i) gel-free methods or (ii) by gel-based methods in combination with mass spectrometric methods. Two-dimensional PAGE can be used to compare different samples. Depending on the characteristics of sub-proteome analyzed, different pH gradients on IPG-strips are available. For example, crude extracts can be separated by IPG/SDS-PAGE (iia) or alternative methods. Off-gel/SDS-PAGE has advantages for proteins with alkaline pI (shown for soluble proteins). Strongly bound cell wall proteins (sCW) are shown as an example for IEF/SDS-PAGE (pH 3–7). Different protein stains allow visualization of either total proteins, glycoproteins or phosphoproteins. Coomassie Colloidal Blue was used for the samples presented in (ii) with exception of (iid). Membrane proteins have to be solubilized before separation and can be analyzed by different 2D-PAGE methods. Analysis by differential in-gel electrophoresis (DIGE) as described by Meisrimler et al. (2011) is shown for plasma membrane samples (iid). Finally, protein gels can be compared using a 2D-PAGE analysis software (e.g., Delta2D). TCA, trichloroacetic acid; gAP, glycosylated proteins of AP; gCW, glycosylated proteins of CW; iCW, ionically bound proteins of CW; PLC, phosphatidylinositol-specific phospholipase C; GPI, glycosylphospho-inositol anchored proteins; MSn, n steps of mass spectrometry; LC, liquid chromatography.

The pea secretome can be divided in at least three sub-proteomes by cell fractionation (Figure 1): (i) apoplastic fluid (ii) cell wall, and (iii) plasma membrane. The cell wall fraction may be further divided into ionically and strongly bound proteins (Jamet et al., 2006). Strongly bound proteins (covalently-bound or bound by interaction with Ca^2+^ pectate) are released by application of cellulase and pectinase from the cell wall matrix. While specific stains in native IEF or 2D-PAGE can detect active enzymes, the high abundance of fungal enzymes used for cell wall digestion may inhibit the MS analysis of proteins with comparable properties. A denaturing sample preparation method has been shown by Chen et al. (2015) for Arabidopsis samples using direct tryptic digestion after digestion of cell walls. Although the addition of cellulase and pectinase can disturb 2D-PAGE due to their high abundance.

Specific cell wall proteins or apoplastic proteins can be also separated by their biochemical properties. *N*-glycosylated proteins can be enriched by lectin affinity chromatography (Minic et al., 2004). Glycoproteins can be identified directly by digestion and MS or separated by PAGE methods followed by glycol staining. Glycoproteomics has not been undertaken in pea, but was successfully applied to its close relative soybean, and was used to analyze the effects of flooding stress on the root glycoproteome (Mustafa and Komatsu, 2014).

The preparation of membranes will separate hydrophobic membrane proteins from soluble proteins. Cell membranes can be further separated by different techniques, e.g., density gradients (sucrose or percoll) for organelles, tonoplast etc. or aqueous polymer two-phase partitioning for plasma membranes (Meisrimler et al., 2011). The glycosylphosphatidylinositol-(GPI)-anchored proteome can be specifically analyzed by cleavage of GPI-anchors by phosphatidylinositol-specific phospholipase C (PLC), which was established for Arabidopsis, but has not been applied to pea as yet (Borner et al., 2003).

In contrast to crude extracts that have been used in many proteomic studies (Table 1), cell fractionation before sample preparation has the advantage of accumulating low abundant proteins. Most of the proteomic approaches shown in Table 1 used two-dimensional SDS-PAGE under denaturing conditions for the analysis of alterations in protein profiles from different samples. The two-dimensional (2D) SDS-PAGE approach is exemplified in Figure 1iia, for crude extracts of control and wounded pea root samples. This figure presents a typical comparison of gels using 2D-analysis software. At the moment, several programs for 2D gel analysis are available, including Delta2D, ImageMaster 2D, Melanie, PDQuest, Progenesis Samespot and REDFIN. The extraction of apoplastic fluid results in small amounts of protein, which appear to be highly problematic for some analysis or detection methods. Thus, in contrast to the crude extracts, 2D-PAGE analysis of apoplastic fluid (Figure 1B) and other sub-proteomes usually reveals fewer protein spots (Meisrimler et al., 2011).

Label and label-free quantitative proteomics are available for both bottom-up and top-down strategies and can be combined also with pre-fractionation approaches. These strategies can be classified as either relative or absolute quantitation methods, however, few studies have been published for pea (Table 1). A typical quantitative proteomics approach is the difference gel electrophoresis (DIGE) (Figure 1iid). DIGE minimal dye labeling was used to study crude extracts, soluble and membrane proteomes of pea in response to elicitation (Amey et al., 2008; Castillejo et al., 2011; Meisrimler et al., 2011). In the past, most quantitative methods used for the studies on pea were semi-quantitative. Multiple gels stained with Coomassie Colloidal Blue or silver were used to quantify specific spots or 2D-PAGE was combined with Western blot analysis (Chen et al., 2009).

A study of the plasma membrane proteome of roots examining iron deficient in pea roots, using DIGE has been conducted (Meisrimler et al., 2011). Additionally, the effect of chitosan treatment on iron deficiency was compared on the protein level. Changes in protein abundance were mainly found in redox proteins from the flavodoxin and flavodoxin-like protein families, copper binding-like proteins, multi-copper oxidases and peroxidases. Aside from DIGE, native IEF-PAGE has been used in combination with in-gel activity staining to estimate changes in ferric-chelate reductase, guaiacol peroxidases and NAD(P)H dependent nitroblue tetrazolium oxidoreductase activities and their dependence on the stressors outlined above.

## Isoenzyme analysis—standard techniques or native approaches

Most proteomic studies on pea used the combination of IPG-strip/SDS-PAGE or native PAGE methods (Table 1). An alternative to the standard IPG-strip-based protein fractionation prioir to PAGE is off-gel fractionation of proteins. An off-gel fractionation for the separation of the microsomal fractions of pea roots and shoots has been published and was shown to be superior when compared to standard IPG-strip separations of hydrophobe and alkaline proteins (Meisrimler and Lüthje, 2012). Other protein fractionation methods include various chromatography methods (e.g., immobilized metal ion affinity chromatography (IMAC), lectin affinity chromatography for glycosylated proteins, size exclusion, ion exchange chromatography, etc.) which can also be used for separation of iso-enzymes. Chromatography separation can be combined with activity profiling, Western blot and MS. Recently, a protocol for phos-tag PAGE (Kinoshita and Kinoshita-Kikuta, 2011), which is an affinity PAGE method for phosphorylated proteins, was demonstrated for the application in targeted pea proteomics (Meisrimler et al., 2015).

Pre-fractionation is especially necessary for class III peroxidases (from the secretory pathway), which have an extremely high number of isoenzymes. Crude extracts will not provide a clear result when examining the regulation of a single isoenzyme (Mika et al., 2010). In targeted proteomics, which focuses on specific proteins or regulations, pre-fractionation strategies can often achieve a much higher resolution than bottom-up techniques alone.

A possibility to increase the resolution of total enzyme activity measurements, which are often used as a standard biochemical approach in physiology, can be accomplished by separation using native or modified PAGE methods. Protocols for these methods have been described recently and can be used for pea (Lüthje et al., 2014). Spectrophotometric measurement allows the estimation of total activities of a cell fraction, whereas separation of native proteins, in accordance to their isoelectric point or molecular mass, enables the discrimination between several isoenzymes in the same fraction. The native IEF-PAGE approach is often used for separation of isoenzymes with close characteristics, as well as for the study of post-translational modifications. For quantification of the activity, various technical replicates (*n* = 7–10) are needed (Mika et al., 2010). The reason for this is not only the gel-to-gel variation, but also the variation in the protein activity itself. Aside from quantification, in-gel activity staining can be used to estimate the differences in the activity profiles between sub-proteomes, differentially treated samples and variable substrates (Meisrimler et al., 2011).

Different staining procedures have been published for peroxidases, Fe and Cu proteins, Fe(III)-chelate reductase, superoxide dismutase etc. (Beauchamp and Fridovich, 1971; Holden et al., 1991; Rothe, 1994; Singh et al., 2005; Meisrimler et al., 2011; Lüthje et al., 2014). Not all staining methods are compatible with modified SDS-PAGE. Some proteins have a higher sensitivity for SDS and comparable detergents (Meisrimler et al., 2015). In these cases, native PAGE replaces the modified SDS-PAGE.

Alterations found by DIGE in plasma membrane proteomes of iron deficient and/or chitosan treated pea roots were supported by in-gel activity stains of the samples after native IEF-PAGE (Meisrimler et al., 2011). This study demonstrated that protein abundance and enzyme activity were altered in the treatment.

## Conclusions

Affinity chromatography and electrophoresis are potential partners to gain insights into sub-proteomes, PTMs and protein functions. Compared to spectrophotometric measurements of total activity a more detailed view on isoenzymes can be realized by activity in-gel staining, which can be combined with MS. This has been shown for cell wall fractions and plasma membranes of pea roots. Fragmentation methods such as electron capture dissociation, electron transfer dissociation and collision-induced dissociation play important roles in proteomics and will also affect the analysis of sub-proteomes. Significant advances in the sensitivity of MS technologies can overcome challenges in pea sub-proteomics, including the analysis of low-abundant proteins or PTMs. Phos-tag in combination with in-gel activity staining will be a powerful tool to study regulation of enzyme activities in orphan species like pea. Finally, future pea proteomics will have to link the numerously existing physiological and biochemical data, with the regulation of single iso-enzymes. This will allow a correlation of regulation of total enzyme activities under various conditions with that of single iso-enzymes. Therefore, the combination of specific in-gel activity staining with MS-analysis needs further improvements.

### Conflict of interest statement

The authors declare that the research was conducted in the absence of any commercial or financial relationships that could be construed as a potential conflict of interest.
